# Characteristics of patients contacting a center for undiagnosed and rare diseases

**DOI:** 10.1186/s13023-016-0467-2

**Published:** 2016-06-21

**Authors:** Tobias Mueller, Andreas Jerrentrup, Max Jakob Bauer, Hans Walter Fritsch, Juergen Rolf Schaefer

**Affiliations:** Center for undiagnosed and rare diseases, University clinic Marburg, Baldinger Str. 1, D-35043 Marburg, Germany; Information technology Department, University clinic Marburg, Baldinger Str. 1, D-35043 Marburg, Germany

**Keywords:** Rare disease epidemiology, Undiagnosed diseases, Undiagnosed disease program

## Abstract

**Background:**

Little is known about the characteristics of patients seeking help from dedicated centers for undiagnosed and rare diseases. However, information about their demographics, symptoms, prior diagnoses and medical specialty is crucial to optimize these centers’ processes and infrastructure.

**Methods:**

Using a questionnaire, structured information from 522 adult patients contacting a center for undiagnosed and rare diseases was obtained. The information included basic sociodemographic data (age, gender, insurance status), previous hospital admissions, primary symptoms of complaint and previously determined diagnosis.

**Results:**

The majority of patients completing the questionnaire were female, 300 (57 %) vs. 222 men (43 %). The median age was 52 years (range 18–92). More than half, 309 (59 %), of our patients had never been admitted to a university hospital. Common diagnoses included other soft tissue disorders, not classified elsewhere (ICD M79, *n =* 63, 15.3 %), somatoform disorders (ICD F45, *n =* 51, 12.3 %) and other polyneuropathies (ICD G62, *n*=36, 8.7 %). The most frequent symptoms were general weakness (*n =* 180, 36.6 %) followed by arthralgia (*n =* 124, 25.2 %) and abdominal discomfort (*n =* 113, 23.0 %). The majority of patients had either internal medicine (81.3 %) and/or neurologic (37.6 %) health problems.

**Conclusions:**

Pain-associated diagnoses and the typical “unexplained” medical conditions (chronic fatigue syndrome, fibromyalgia, irritable bowel syndrome) are frequent among people contacting a center dedicated to undiagnosed diseases. The chief symptoms are mostly unspecific. An interdisciplinary organizational approach involving mainly internal medicine, neurology and psychiatry/psychosomatic care is needed.

## Background

Determining the correct diagnosis of a rare and undiagnosed disease is sometimes troublesome. The diagnostic possibilities of undiagnosed diseases include the following: (1) a rare disease, (2) an unusual presentation of a more common disease, (3) the simultaneous occurrence of multiple diseases, and (4) an underlying truly new disease. In particular, ultra-rare diseases are challenging because standard diagnostic algorithms may not cover these diseases due to their low prevalence. Often, patients involve a number of physicians from different specialties and are admitted multiple times to different hospitals with little communication between institutions. At some point, even the most skillful physician begins to question his clinical reasoning and/or the patient’s reliability. Extensive diagnostic tests will be repeated, imaging studies conducted, and altogether, the physician-patient relationship suffers. Moreover, treatment delays and increased healthcare costs occur as a consequence. However, it is important to state that rare diseases are not always undiagnosed, and undiagnosed diseases are not always rare.

Not every undiagnosed or rare disease presents a challenge for the general physician. Some diseases present with pathognomonic, very characteristic symptoms, and thus an instant diagnosis can be made by simply looking at the patient, conducting a thorough physical examination or performing a simple test.

Other diseases present with characteristic combinations of symptoms that are quite common on their own but are pathognomonic in their unique combination. These are the famous triads or tetrads of symptoms that are laboriously memorized by every medical student at some point in their education. For example, Heerfordt’s syndrome, a rare form of sarcoidosis, presents with a combination of uveitis, facial palsy and parotitis. Flush, diarrhea and cardiac symptoms point to endocrine tumors. Iritis, oral and genital aphthous lesions and erythema nodosum are typical of Behcet’s disease. For these entities, a diagnosis can be determined by simply entering the symptoms into a standard web search engine. For genetic diseases, more specialized search engines are available [1].

The geographical aspects of a disease play a critical role as well. This is especially true for rare infectious diseases. In western society, common infectious diseases such as schistosomiasis, Q fever and tuberculosis are currently quite uncommon. Rocky Mountain spotted fever and Chagas are only endemic in certain regions. Consequently, physicians aside from those in tropical medicine are usually unfamiliar with the symptoms and diagnosis of these diseases. Furthermore, patients neglect the health risks of traveling long distances at the last minute. However, “infectious diseases do not need to buy plane tickets”; in effect, they travel for free with infected patients. Accordingly, given the high international mobility of patients and the rapid same-day logistics of acquiring goods, even a rural-based western family physician can encounter almost any infectious or tropical disease. This is especially true during days of high migration.

Diseases that present with unspecific, common symptoms such as indigestion, nausea, dizziness, joint pain or headache are difficult to distinguish. The differential diagnosis of these symptoms is usually broad. On the one hand, these are quite often symptoms of a general illness or adverse effects of the patients’ medications. On the other hand, these symptoms can be signs of serious diseases as well. This could lead to a cost-intensive testing of a variety of different lab values. Although broad testing might often be unnecessary and counterproductive, it is sometimes justified when otherwise frequent physician and hospital visits could be avoided [2].

The diagnostic heuristics for rare and undiagnosed diseases are the same. They both require broad interdisciplinary engagement, access to modern information technology and knowledge resources, and special laboratory diagnostic possibilities including molecular genetics and imaging facilities. Therefore, it is useful to establish an interdisciplinary center of expertise at a tertiary university hospital to provide patients and physicians a central point of care.

The first systematic program for diagnosing rare and obscure diseases was the Undiagnosed disease program established by the National Institutes of Health (NIH) in 2008 [3–5]. The NIH Undiagnosed Diseases Program (UDP), supported by the Office of Rare Diseases Research, the National Human Genome Research Institute (NHGRI), and the NIH Clinical Center, was established to diagnose patients who have long sought a diagnosis and to discover new diseases and insights into their physiology, cell biology, and biochemistry. As there is a substantial need for centers for undiagnosed patients, both from a patient as well as from a physician perspective, multiple national programs and the Undiagnosed Diseases Network International (UDNI) were established [6]. Patients with complex symptoms often consult multiple, different physicians and are intense users of the healthcare system. In many cases, after numerous tests and procedures, this leads to psychiatric or psychosomatic diagnoses, which are often unsatisfactory. Fibromyalgia is a typical diagnosis that is often over-diagnosed in chronic pain patients even if they do not sufficiently fulfill the diagnostic criteria [7, 8]. However, its distinction from Dercum’s disease, a rare disease, is sometimes arbitrary, especially in cases when the patient is obese [7, 9]. Additionally, physicians need a center to refer their patients to when the cascade of diagnostics and referrals is complete.

In December 2013, a center for undiagnosed and rare diseases was established at the University Clinic of Marburg, Germany. The center assists patients and physicians with diagnostic proposals. The inquiries are generally patient initiated, and the center is open to the general public. However, requests from physicians are preferentially considered. All patients can send their patient history accompanied by a letter describing the leading symptoms in their own words to request our proposal. The service is free of charge. After the file with the patient’s history is received, it is prepared to be presented at weekly rounds to experts from all medical fields. Comparable to the classical tumor board approach, the case is presented to a board consisting of experienced senior staff members from different specialties: family medicine, nephrology, rheumatology, gastroenterology, cardiology, endocrinology, pneumology, hematology, laboratory chemistry, neurology, psychosomatic and radiology. Each case will then be discussed for approximately 15 to 30 min; in sum, with 10 participating team members, this leads to a “Physician Brain-Time” of 2.5–5 h. In daily consultations, this amount can hardly be achieved, even if the patient is sent to 10 separate specialists. Furthermore, the intellectual exchange is often more intense and the differential diagnostic aspects more diverse than when on an individual basis. Afterwards, a prospective differential diagnosis and recommendations for further testing are sent to the patient’s physician based on the case discussion. For our center, this system is considered optimal, as it is both practical and economically feasible to tackle complex cases. In this regard, our approach clearly differs from those of the US UDP and UDNI, as in those programs, applications are initially evaluated by a multi-specialty board based on objective signs, symptoms and characteristics that suggest a reasonable likelihood of obtaining a diagnosis with current and imminent technologies. Afterwards, patients are comprehensively evaluated directly at their respective clinical sites [5]. Most of the cases assessed at our center appear to be rather common. However, some of them are also obscure, such as depression caused by a contraceptive hormone spiral, migraine as a result of a breast implant, cobalt intoxication from a defective hip implant [10], partial Sheehan’s syndrome after complications during birth, allergic alveolitis from parrots and scurvy caused by an “acid-free” diet. All of these patients had been undiagnosed for many years. Often, social factors (job, travel, hobbies) identified by intensive anamnesis provided the critical clue needed for the final diagnosis.

However, little is known to date on the characteristics of the patients seeking help from these dedicated centers. As this information is crucial for all efforts aiming to optimize these types of centers (i.e., what experts are needed and what structure must be provided), we analyzed the most important characteristics of more than 500 of our patients.

## Methods

From December 2013 to July 2014, we sent out 703 questionnaires to patients (418 female, 285 male) to obtain structured information on their medical history. The questionnaire asked for basic sociodemographic data (age, gender, insurance status) as well as the primary symptoms of their complaint (in free text). In addition, patients were asked to list the diagnoses that had previously been made. To assess the use of the healthcare system, the number of general and university hospital stays in the past three years was reported on an ordinary scale. A total of 522 (74 %) questionnaires were returned and are included in this analysis. The questionnaires were reviewed by three different readers. These readers looked for key words among the symptom descriptions, and every symptom of complaint was coded using an appropriate SNOMED CT term that provided the closest match. The symptoms were aggregated and subsequently grouped. The diagnoses were coded using the latest ICD-10 German Modification classification by an experienced physician. To assess the primary specialties involved, each patient was assigned a maximum of three specialties based on their detailed description of symptoms and previous diagnoses. Already diagnosed rare diseases were afterwards identified by comparing the coded ICD diagnosis against a list of diagnoses classified as rare diseases based on the European definition of a prevalence lower than 1 per 2000 persons [11].

The datasets were saved in a Microsoft access database, Version 2013. Statistical analyses were performed using SAS Studio software, university edition, Version 3.4, Cary, North Carolina. Wilcoxon-Mann–Whitney *U*-test was used for the comparison analysis (i.e., if gender or insurance status influenced general healthcare usage) and to assess the number of extracted symptoms. To test for gender-related differences in the previously received diagnoses and reported symptoms, *χ*^2^ tests were performed. In all cases, a p value below 0.05 was considered statistically significant. Prior informed consent was obtained from all patients, and ethical approval of the study was obtained from the universities’ local ethical committee.

## Results

**Fig. 1:**
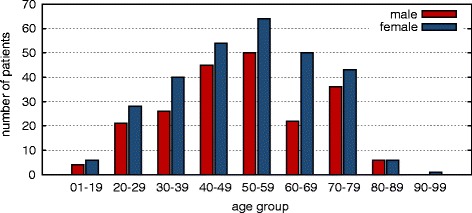
Age of patients grouped by decade and gender (male: blue; female: red)

**Fig. 2:**
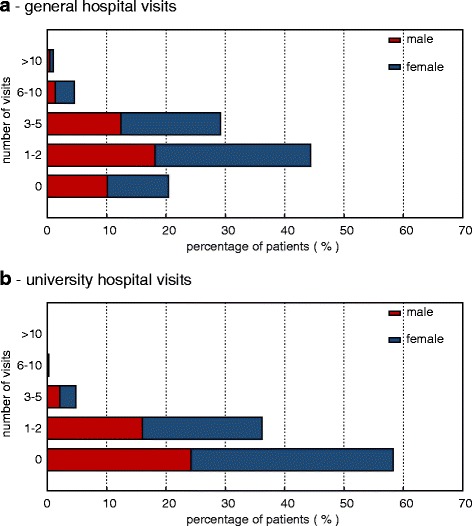
Visits to general and university hospitals, grouped by frequency and gender (male: blue; female: red) in the last three years

The majority of patients returning the questionnaire were female, 300 (57 %) vs. 222 men (43 %). The median age was 52 years (range 18–92), with the majority of patients being in their 6th decade (50–59 years); see Fig. 1 for details. Age did not significantly differ between these two groups. Of all patients, 448 (86 %) were covered by statutory health insurance, 69 (13 %) were covered by private insurance and 5 patients (1 %) had an unknown insurance status. As the proportion of privately insured patients in the German population is 11 %, privately insured patients were only marginally overrepresented in our cohort [12]. The majority of our patients (233, 44 %) had received one to two stays in a general hospital, and 309 (59 %) had never been admitted to a university hospital before (see Fig. 2). Healthcare usage as measured by the number of general or university hospital stays was not significantly different between men/women or statutory/private health insured patients (all *p >* 0.05). Additionally, 51 % of all patients (53 % female, 47 % male, *p =* 0.2069) had consulted an alternative practitioner before contacting our center. Most patients had learned about our center in the media (print media 32.3 %; internet 15.2 %, television 36.9 % and other 15.6 %) following our publication of a cobalt intoxication due to an artificial hip transplant in The Lancet [10].

Of all 522 received questionnaires, 413 patients (242 female, 171 male) included information about a prior diagnosis. The patients listed a median of 3 diagnoses (range 1–20). Conditions grouped by ICD code M79 – other soft tissue disorders, not classified elsewhere (including fibromyalgia, neuralgia, myalgia and rheumatism) were significantly more common among female patients. This group included the single most reported diagnosis of fibromyalgia (M79.7), reported by 36 (9 %) of our patients. Fibromyalgia was significantly more frequent in women (26 female, 7 male, *p =* 0.0051). As the group was heterogeneous, it should be noted that panniculitis (M79.3), hypertrophy of the infrapatellar fat pad (M79.4), and residual foreign body in soft tissue (M79.5) did not occur. Conditions grouped by ICD code M35—other systemic involvement of connective tissue, such as sicca syndrome, overlap syndrome, and polymyalgia rheumatica, were significantly more common among women as well. The only condition that was significantly more frequent among male patients was sleep apnea syndrome, listed under ICD code G47. Overall, the high prevalence of pain-related diagnoses is evident. In total, 51 patients (28 female, 23 male) contacted us with a diagnosis classified as a rare disease condition. Of these, the largest subgroup represents 12 patients with known or highly suspected motor neuron disease (11 amyotrophic lateral sclerosis, 1 bulbar palsy). Table 1 summarizes the 15 most frequent ICD diagnoses by category.

**Table 1:** Top 15 ICD diagnosis

			Total (*n =* 413)		Men (*n =* 171)		Women (*n =* 242)		
Rank	ICD	ICD Caption	(n)	(%)	(n)	(%)	(n)	(%)	*χ* ^2^ p-value
1	M79	Other soft tissue disorders, not elsewhere classified (incl. fibromyalgia, myalgia, neuralgia, rheumatism)	63	15.3 %	16	9.4 %	47	19.4 %	0.0051
2	F45	Somatoform disorders	51	12.3 %	18	10.5 %	33	13.6 %	0.3440
3	G62	Other polyneuropathies (incl. alcoholic, drug induced, toxic, unspecified polyneuropathies)	36	8.7 %	17	9.9 %	19	7.9 %	0.4582
4	M54	Dorsalgia	30	7.3 %	9	5.3 %	21	8.7 %	0.1879
5	I10	Essential (primary) hypertension	29	7.0 %	15	8.8 %	14	5.8 %	0.2420
6	F32	Depressive episode	28	6.8 %	11	6.4 %	17	7.0 %	0.8136
	M35	Other systemic involvement of connective tissue (incl. sicca syndrome, overlap syndrome, polymyalgia rheumatica)	28	6.8 %	5	2.9 %	23	9.5 %	0.0088
	A69	Other spirochetal infections (incl. Lyme’s disease)	28	6.8 %	16	9.4 %	12	5.0 %	0.0799
7	K58	Irritable bowel syndrome	25	6.1 %	12	7.0 %	13	5.4 %	0.4897
8	T78	Adverse effects, not elsewhere classified (incl. food allergies)	24	5.8 %	8	4.7 %	16	6.6 %	0.4082
9	G93	Other disorders of brain (incl. postviral fatigue syndrome)	21	5.1 %	10	5.8 %	11	4.5 %	0.5529
10	K29	Gastritis and duodenitis	20	4.8 %	5	2.9 %	15	6.2 %	0.1268
11	M19	Other arthrosis	19	4.6 %	6	3.5 %	13	5.4 %	0.3733
12	M47	Spondylosis	18	4.4 %	4	2.3 %	14	5.8 %	0.0911
13	G47	Sleep disorders (incl. sleep apnea)	17	4.1 %	13	7.6 %	4	1.7 %	0.0027
14	E03	Other hypothyroidism	16	3.9 %	7	4.1 %	9	3.7 %	0.8460
	G25	Other extrapyramidal and movement disorders (incl. restless legs syndrome)	16	3.9 %	10	5.8 %	6	2.5 %	0.0806
	M51	Other intervertebral disc disorders	16	3.9 %	6	3.5 %	10	4.1 %	0.7464
15	E55	Vitamin D deficiency	14	3.4 %	7	4.1 %	7	2.9 %	0.5065

The 15 most frequent ICD diagnosis previously received by our patients grouped by gender and ICD three-letter code. In cases where multiple other diagnoses are grouped together, the specific diagnostic terms are included

**Fig. 3:**
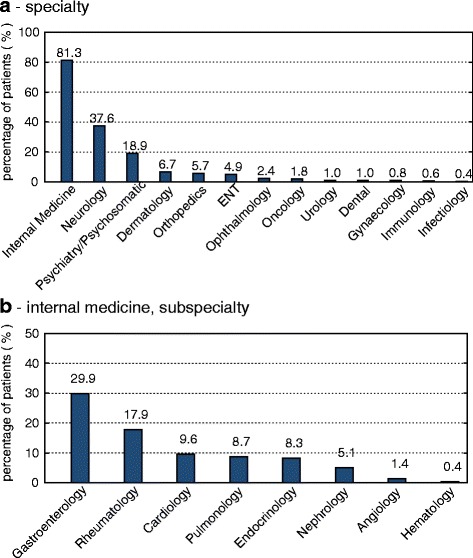
Classification of case presentations by specialty (a); as the largest number was assigned to internal medicine, this group was further divided by subspecialty (b)

Furthermore, 492 patients included a detailed description of their symptoms and ailments in the questionnaire (286 female, 206 male). The median number of extracted symptoms was 4 (range 1–25). Female patients described their symptoms in more detail, meaning significantly more symptoms could be extracted from their descriptions (*p =* 0.0175). The most frequent symptom was a complaint of general weakness and increased fatigue, followed by arthralgia and abdominal discomfort and pain. A detailed list of the 20 most common symptoms is provided in Table 2. Clearly, pain-related symptoms were the leading complaints of most patients. Based on the symptom descriptions, the majority of patients were assigned as having internal medicine (mostly gastroenterological or rheumatological complaints) and neurological health problems (see Fig. 3 for details).

**Table 2:** Top 20 symptoms

		Total (*n =* 492)		Men (*n =* 206)		Women (*n =* 286)		
Rank	Symptom	(n)	(%)	(n)	(%)	(n)	(%)	*χ* ^2^ p-value
1	Asthenia, general weakness, increased fatigue	180	36.6 %	69	33.5 %	111	38.8 %	0.2271
2	Arthralgia	124	25.2 %	50	24.3 %	74	25.9 %	0.6863
3	Abdominal discomfort and pain	113	23.0 %	37	18.0 %	76	26.6 %	0.0251
4	Headache	91	18.5 %	25	12.1 %	66	23.1 %	0.0020
5	Back pain	84	17.1 %	26	12.6 %	58	20.3 %	0.0259
6	Full body pain	82	16.7 %	31	15.0 %	51	17.8 %	0.4137
7	Dizziness	81	16.5 %	30	14.6 %	51	17.8 %	0.3347
8	Localized pain in a single extremity	79	16.1 %	36	17.5 %	43	15.0 %	0.4669
9	Generalized myalgia	76	15.4 %	30	14.6 %	46	16.1 %	0.6452
10	Gait difficulties	73	14.8 %	33	16.0 %	40	14.0 %	0.5313
11	Paresthesia and dysesthesia	61	12.4 %	23	11.2 %	38	13.3 %	0.4811
12	Muscle cramps and spasms	59	12.0 %	21	10.2 %	38	13.3 %	0.2975
13	Nausea and vomiting	55	11.2 %	12	5.8 %	43	15.0 %	0.0014
14	Palpitations, heart rhythm abnormalities	54	11.0 %	21	10.2 %	33	11.5 %	0.6379
15	Visual disorder, disturbance or defects	53	10.8 %	25	12.1 %	28	9.8 %	0.4077
16	Abnormal stool consistency and frequencies	51	10.4 %	17	8.3 %	34	11.9 %	0.1918
17	Dyspnea	43	8.7 %	16	7.8 %	27	9.4 %	0.5167
18	Dyssomnia	39	7.9 %	16	7.8 %	23	8.0 %	0.9113
	Sensation of abnormal heat or cold	39	7.9 %	18	8.7 %	21	7.3 %	0.5720
19	Edema	37	7.5 %	8	3.9 %	29	10.1 %	0.0094
20	Hyperhidrosis	34	6.9 %	14	6.8 %	20	7.0 %	0.9323
	Exanthema, erythema	34	6.9 %	13	6.3 %	21	7.3 %	0.6561

Absolute and percentage of the 20 most frequent symptoms grouped by gender

## Discussion

As evidenced by the increasing number of specialized centers and programs, undiagnosed and rare diseases are the focus of current developments in the health sector. This is due to the higher degree of specialization in almost all fields of medicine, focusing mostly on procedural, well-refunded services rather than poorly funded diagnostics [13]. Thus far, little is known about the characteristics of the patients contacting such centers. This study provides detailed insight into this group of patients. These results are important for optimizing the structure and procedures of centers dedicated to helping this suffering group of patients.

The large number of patients seeking some type of support from centers such as ours underlines the need for high-end diagnostic institutions. The raw figures of inquiries are comparable with those of the NIH Undiagnosed Diseases Program; however, nothing compares with the prestigious NIH, which is of course also true for our limited personnel and financial resources [4, 5, 14, 15].

Age and gender are the two most significant predisposing factors for illness and disease. The majority of patients were in their sixth decade of life (50–59 years). The predominance of females among our patients is again in accordance with the NIH’s figures and could be due to various reasons. The most obvious reason is that the adult female population (age 18 years and older) in Germany outnumbers the male population by approximately 6 % [16]. Thus, a female dominance in this range is expected purely by demography. Another factor contributing to this observation might be that women tend to use ambulatory healthcare services in industrialized societies more frequently than men do [17], which is also true for Germany [18–20]. A potential bias might be the broad media coverage on our center. Because women are more active in seeking health information, this might have led to an increase in contacting our center [21–23].

Interestingly, only 41 % of our patients had been referred to another university hospital before their initial contact with our center. In contrast, 51 % of our patients had consulted an alternative practitioner. This is surprising and indicates that alternative practitioners are easier to access than academic centers. This presents an obvious problem because university hospitals, with their comprehensive diagnostic capabilities and their multidisciplinary structure, are an optimal environment for complex and challenging cases. This points to a failure in the referral system and highlights that the regular diagnostic pathway from general practitioner to specialist to university center is not functioning properly, which leaves numerous patients on their own.

The prevalence of pain-associated diagnoses is generally high among our patients. In particular, unspecified soft tissue disorders (ICD M79), characterized by pain in the joints, muscles, nerves, limbs or even the whole body, is reported by 15 % of our patients. Altogether, the prevalence of chronic pain conditions is high, the healthcare usage and associated costs are high, and the number of consulted physicians is high [24–27]. Because our center is specialized to rare and/or unexplained diseases, most patients who were referred to us suffered from medically “unexplained” conditions (e.g., fibromyalgia, irritable bowel syndrome, chronic fatigue syndrome) [28, 29]. The high prevalence of somatoform and affective disorders can be seen in this context. In addition, numerous patients with rare diseases contacted our center (*n =* 51; 12 %). Among those, patients with known amyotrophic lateral sclerosis (ALS) comprised the largest subgroup (*n =* 12, 24 %). Because the diagnosis of ALS had already been made, these patients did not need our center to achieve a final diagnosis; however, they clearly hoped for help in confronting this—so far—devastating disease. In contrast, the fatality of a disease seemed not to be a considerable factor, as, e.g., only a few patients with progressive neoplasms contacted us. However, this might be due to the numerous support systems that exist as “second opinion centers” for cancer patients.

The majority of health complaints were subjective, with fatigue, arthralgia and abdominal discomfort listed as the three most common symptoms. The high prevalence of unspecific health complaints especially in the musculoskeletal, neurological and gastrointestinal system has been identified in broad population studies from health insurance and healthcare providers [30, 31]. Comparable to our observation of a median of 4 reported symptoms, these studies report a median number of symptoms ranging from 4–8. However, the methodology in these studies differed from our own. Instead of selecting symptoms from a predefined list of symptoms, we used an open-ended free-text approach comparable to an initial question in a traditional anamnesis.

Most of our patients had conditions in the area of internal medicine. Internal medicine can further be divided into subspecialties, and neurology was the most prominent single category. Due to the high comorbidity with suspected psychosomatic disorders, this specialty is similarly over-represented. Interestingly, our patient characteristics are comparable with those reported by Gahl et al. for the NIH UDP [15], with large numbers of neurological and gastrointestinal cases. Nevertheless, as our evaluation process differs from theirs and the vast majority of our cases are self-referrals, these statistics are difficult to compare. These data are important for the structure and composition of interdisciplinary teams in centers dedicated to undiagnosed and rare diseases in adults. In this cohort, neurological and gastrointestinal cases were prominent as well. The assignment of these complex cases to a single specialty is not possible, as they often have multiple diagnoses and symptoms involving various organ systems and disciplines. Therefore, not only a multidisciplinary but a true interdisciplinary approach is essential.

The main limitation of our study was the reliance on self-reported data for all the variables of investigation. The accuracy of self-reported healthcare usage is strongly influenced by the timeframe and actual frequency. A longer timeframe could lead to underestimation [32]. As we used an open-end free-text approach to study the main symptoms, factors such as education, clinical experience and other factors might bias the findings. As our center only offers its service to adults, genetic diseases that have onsets in childhood are unrepresented. At this point in time we are unable to report a success rate of our approach, which is the topic of a follow up study.

Our initial data demonstrate a substantial need in our healthcare system for dedicated centers for patients with unclear and undiagnosed diseases. For these patients (and for us), it is unimportant whether their request is objectively, medically justified—these patients suffer and feel undiagnosed. Usually multiple physicians had been previously involved, a previous diagnosis of one of the classical “unexplained” medical conditions established, and the number of “unspecific” symptoms high. These patients search for an underlying somatic explanation. With increasing research, there is rising evidence that a number of rare conditions can mimic these “unexplained” conditions. For instance, the improved understanding of rare hereditary sodium channelopathies suggests that these may play a vital role in the diagnosis and treatment of some painful neuropathies [33, 34] and that in some cases, the initial diagnosis of fibromyalgia was misleading [35]. Other rare conditions prone to delayed diagnosis are adrenal insufficiency, hereditary angioedema and cerebrotendinous xanthomatosis [35–38]. A new cornerstone could be the identification of novel possibilities along with the broad availability of next-generation-sequencing (NGS). The initial results lead to promising insights [39, 40]. The increasing use of clinical decision support systems and dedicated software systems will improve our approach regarding undiagnosed diseases [41].

## Conclusion

There is a high demand by adult patients who feel undiagnosed and who are seeking a second opinion in a specialized center. Therefore, dedicated centers for undiagnosed and rare diseases are needed. The majority of patients were middle-aged, with a slight female predominance. Pain-related diagnoses and conditions were frequent, along with diagnoses including the typical “unexplained” medical conditions (chronic fatigue syndrome, fibromyalgia, irritable bowel syndrome). The chief symptoms were unspecific in general. An interdisciplinary organizational approach involving mainly internal medicine, neurology and psychiatry/psychosomatic is needed.

## Abbreviations

ALS, amyotrophic lateral sclerosis, ICD, international classification of diseases, NHGRI, National Human Genome Research Institute, NIH, National Institutes of Health, UDNI, Undiagnosed Diseases Network International, UDP, Undiagnosed Diseases Program, US, United states, SNOMED CT, Systematized Nomenclature of Human and Veterinary Medicine Clinical Terms
